# Clinical Diagnosis of Chikungunya Infection: An Essential Aid in a Primary Care Setting Where Serological Confirmation Is Not Available

**DOI:** 10.3390/tropicalmed8040213

**Published:** 2023-04-03

**Authors:** Juan C. Rueda, Ingris Peláez-Ballestas, Jose-Ignacio Angarita, Ana M. Santos, Carlos Pinzon, Eugenia-Lucia Saldarriaga, Jorge M. Rueda, Elias Forero, Diego L. Saaibi, Paula X. Pavía, Marta Juliana Mantilla, Gustavo Rodríguez-Salas, Juan Camilo Santacruz, Igor Rueda, Mario H. Cardiel, John Londono

**Affiliations:** 1 Biosciences Programme, Faculty of Medicine and Engineering, Universidad de La Sabana, Chía 53753, Colombia; 2 Grupo de Espondiloartropatías, Rheumatology Department, Universidad de La Sabana, Chía 53753, Colombia; 3 Rheumatology Unit, Hospital General de México “Doctor Eduardo Liceaga”, Mexico City 06729, Mexico; 4 Departamento de Investigación Clínica, Facultad de Medicina, Universidad de La Sabana, Chía 53753, Colombia; 5 Rheumatology Unit, Centro Médico Imbanaco, Universidad Libre, Cali 760042, Colombia; 6 Rheumatology and Internal Medicine Department, Universidad del Norte, Barranquilla 081007, Colombia; 7 Reumatología Ubit, Centro Médico Carlos Ardila Lulle, Bucaramanga 681004, Colombia; 8 Unidad de Investigación Científica, Hospital Militar Central, Bogotá 110231, Colombia; 9 Rheumatology Department, Hospital Militar Central, Bogotá 110231, Colombia; 10 Centro de Investigación Clínica de Morelia SC, Morelia 58280, Mexico

**Keywords:** Chikungunya virus, diagnosis, arbovirus infections, clinical decision making, Colombia

## Abstract

Background: Chikungunya virus (CHIKV) diagnosis has become a challenge for primary care physicians in areas where the Zika virus and/or Dengue virus are present. Case definitions for the three arboviral infections overlap. Methods: A cross-sectional analysis was carried out. A bivariate analysis was made using confirmed CHIKV infection as the outcome. Variables with significant statistical association were included in an agreement consensus. Agreed variables were analyzed in a multiple regression model. The area under the receiver operating characteristic (ROC) curve was calculated to determine a cut-off value and performance. Results: 295 patients with confirmed CHIKV infection were included. A screening tool was created using symmetric arthritis (4 points), fatigue (3 points), rash (2 points), and ankle joint pain (1 point). The ROC curve identified a cut-off value, and a score ≥ 5.5 was considered positive for identifying CHIKV patients with a sensibility of 64.4% and a specificity of 87.4%, positive predictive value of 85.5%, negative predictive value of 67.7%, area under the curve of 0.72, and an accuracy of 75%. Conclusion: We developed a screening tool for CHIKV diagnosis using only clinical symptoms as well as proposed an algorithm to aid the primary care physician.

## 1. Introduction

Chikungunya virus (CHIKV) is a member of the Semliki Forest virus antigenic complex and is classified as an alphavirus from the *Togaviridae* family, which causes acute arthropathy in humans, similar to other alphaviruses [1]. After the epidemic in La Reunion in 2006, due to an adaptive mutation of alanine for valine at position 226 (A226V) in the E1 glycoprotein of CHIKV, it gained the ability to be transmitted not only by *Aedes (Ae) aegypti* but also by *Ae. albopictus* [2].

**Figure 1:**
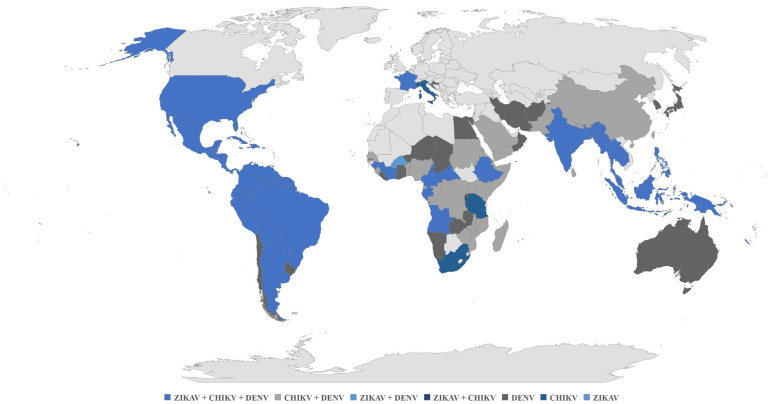
World distribution of DENV, CHIKV and ZIKAV. DENV: Dengue virus; CHIKV: Chikungunya virus; ZIKAV: Zika virus [7,8,9].

The Asian lineage of CHIKV rapidly spread to the Western Hemisphere, affecting 42 countries by 2015 and finally reaching Colombia in August 2014 after arriving at the Island of Saint Martin in 2013 [3,4,5,6]. CHIKV infection became a worldwide spread epidemic, affecting countries where other arboviral diseases, for example, infections caused by the Zika virus (ZIKAV) or Dengue virus (DENV), were present (Figure 1). 

**Figure 2:**
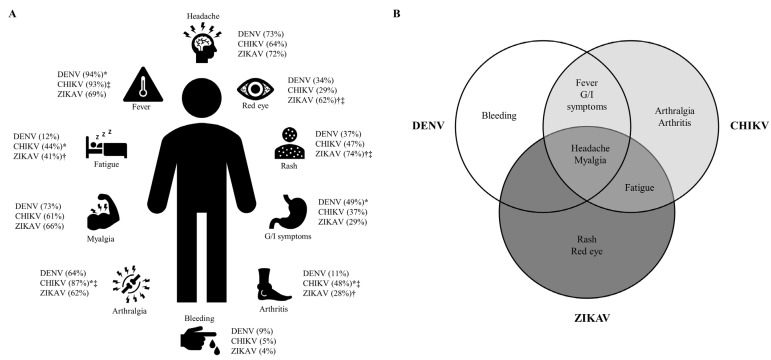
Common symptoms in arboviral infection (DENV, CHIKV and ZIKAV). Shown are the common symptoms in DENV, CHIKV and ZIKAV. In panel (**A**) are the percentages and statistical significance (*p* < 0.05) of each symptom between DENV-CHIKV (*), DENV-ZIKAV (†) and CHIKV-ZIKAV (‡). In panel (**B**) are depicted the shared and single statistically significant symptoms between CHIKV, DENV and ZIKAV. See supplementary Tables S1 and S2 for references. DENV: Dengue virus; CHIKV: Chikungunya virus; ZIKAV: Zika virus.

According to the Centre for Disease Control and Prevention data, half of the countries with a previous arboviral infection have reported autochthonous transmission of the three viruses [7,8,9]. In these countries, diagnosis in a primary care setting becomes a challenge since the infections caused by CHIKV, DENV, and ZIKAV share clinical symptoms. Fever, headache, myalgia, and bleeding are frequently reported symptoms in patients suffering from CHIKV, ZIKAV, or DENV infection [10,11,12,13,14,15,16,17,18,19,20,21,22,23,24,25,26,27,28,29,30]. However, some symptoms are more specifically associated with each virus; for example, arthralgia and arthritis in CHIKV infection, rashes and red eyes in ZIKAV infection, and fever and gastrointestinal symptoms in DENV infection (Figure 2, Tables S1 and S2).

Therefore, diagnosis of CHIKV infection requires laboratory confirmation by a polymerase chain reaction (PCR), serological test, or viral culture [31]. Directing public health care policies, confirming a clinical diagnosis, and conducting accurate infectious disease surveillance requires proper laboratory testing; however, access is restricted in many middle- or low-income tropical or subtropical countries, especially where primary care physicians face this infection [11,32]. According to reports from the National Health Institute, in Colombia, only 1.08% of CHIKV-infected patients (5231 out of 482,326) were clinically confirmed in a laboratory between epidemiological week 37 of 2014 and week 44 of 2019 [33,34,35,36,37,38]. The lack of confirmation of CHIKV infection increases the need for a reliable clinical diagnostic tool to aid primary care physicians when facing patients where common arboviral diseases caused by CHIKV, ZIKAV, and DENV are endemic or epidemic. 

For this reason, we decided to evaluate the performance of the currently used diagnostic criteria for CHIKV infection. We applied the criteria for improvement to a population with confirmed CHIKV infection and, with the help of expert consensus, we created a diagnostic screening tool based on clinical symptoms.

## 2. Materials and Methods

### 2.1. Study Population

A cross-sectional analysis was conducted in community cohorts from Bogotá, Cali, Medellin, Cúcuta, Bucaramanga, and Barranquilla (Colombia). The included cities were chosen to represent the Colombian population, and the included patients between 2014 and 2015 were aged ≥18 years. The Community Oriented Program for Control of Rheumatic Diseases (COPCORD) methodology was used to include patients in the study [39]. COPCORD is an economical program that evaluates and measures disability and pain from rheumatic diseases. It is designed to be implemented in communities of developing countries. In 2014, from August to September, the CHIKV epidemic struck the country while the Colombian COPCORD study was being developed. Since CHIKV infection is mainly associated with musculoskeletal (MSK) symptoms, CHIKV-infected patients had to be identified within the studied population to avoid an increase in cases in the COPCORD study. Information on socioeconomic and sociodemographic variables such as age, gender, ethnicity, origin, monthly income, and socioeconomic strata were collected using a questionnaire. Individuals were asked about non-traumatic MSK symptoms, such as stiffness, pain, arthralgia, or arthritis. A patient was considered COPCORD-positive if any of these symptoms were present at any moment in their life or 7 days prior to the interview. Every COPCORD-positive patient was questioned regarding CHIKV infection symptoms. If CHIKV infection was considered, a secondary examination was carried out within the following 7 days by a trained rheumatologist or rheumatology fellow. The criteria for suspected cases of CHIKV fever were applied according to the World Health Organization (WHO) guidelines [31]. Blood samples were collected from patients who were asked about their joint, gastrointestinal, and dermatological symptoms using a specifically designed questionnaire. Patients were assessed once and were factored out when a rheumatic disease was suspected or confirmed by the physician. All samples from the suspected patients, when analyzed for DENV-specific IgM antibodies, produced a negative result. At the time of data collection, the ZIKAV epidemic was not present in Colombia. 

### 2.2. WHO CHIKV Infection Case Definition [31]

A case was suspected based on epidemiological criteria (living or visiting geographical areas with reports of transmission within 15 days prior to the onset of symptoms) and clinical criteria (acute onset of high temperature >38.5 °C and “incapacitating joint pain”). A confirmed case was considered when the presence of virus-specific IgM or IgG antibodies were demonstrated, irrespective of the clinical presentation or stage of the disease. On the grounds that our patients had no previous reports of CHIKV infection before this epidemic, and were therefore immunologically naïve, we took into account the presence of virus-specific IgG antibodies in a single serum sample at any point of the disease as positive confirmation of a CHIKV infection.

### 2.3. CHIKV Serological Analysis

Enzyme-linked immunosorbent assay (ELISA) with chikungunya IgG and IgM antibodies was performed according to the manufacturer’s guidelines (ab177848 anti-CHIKV IgM and ab177835 anti-CHIKV IgG, Abcam, Cambridge, UK). 

### 2.4. Statistical Analysis

The mean and standard deviation (SD) were used for continuous variables and counts while percentages for categorical variables were used for descriptive analyses. Two-by-two tables were used to establish associations between categorical variables. Student’s *t*-test was used to compare the mean values. Statistical significance was set at *p* < 5%. For associations, odds ratios (ORs) were calculated with 95% confidence intervals (CIs). A positive CHIKV serology result (IgG or IgM) was used to identify subjects with CHIKV infections. Bivariate analysis was performed, including all studied variables, using confirmed CHIKV infection as the outcome. Variables with significant statistical association with the outcome were included by consensus. Agreed variables were analyzed in a multiple regression model using a stepwise forward method. Hosmer and Lemeshow’s goodness-of-fit test was used to assess model performance. The area under the receiver operating characteristic (ROC) curve was calculated to determine the cut-off value and performance. SPSS (Statistical Package for the Social Sciences; version 22.0; IBM, Armonk, NY, USA) was used for data analysis. 

### 2.5. Agreement Consensus

Specialists from different regions of Colombia with experience in diagnosing and treating CHIKV infection (five rheumatologists, two epidemiologists, and two tropical medicine specialists) met face-to-face to conduct an agreement study on the clinical characteristics of CHIKV infection and its associations. Sequential questions were prepared and answered in real-time to determine which statistically significant variables obtained from the bivariate analysis should be considered clinical criteria for CHIKV diagnosis (see Table 3 for details on the questionnaire). Only the following answers were possible: totally agree, agree, not in agreement or disagreement, disagree, and totally disagree. Answers were calculated as percentages, and a percentage ≥ 50% was set as agreement, regardless of the answer. When agreement was not reached, the moderator reformulated the question after discussing the opinions of conflicting members. This procedure was repeated until a consensus was reached. 

## 3. Results

### 3.1. Study Participants

**Figure 3:**
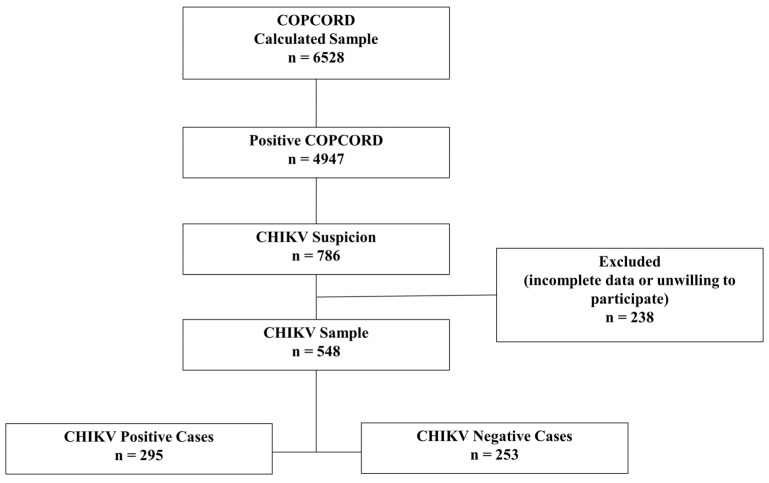
Profile of the study population. COPCORD: Community Oriented Program for Control of Rheumatic Diseases; CHIKV: Chikungunya virus.

In the COPCORD study, 6528 people were surveyed in their homes. Of these, 548 have been included in the present study due to clinical suggestions of CHIKV infection. All 548 subjects were serologically tested for CHIKV antibodies to confirm the diagnosis, and 295 (53.8%) resulted as being positive for IgG or IgM (Figure 3).

### 3.2. Demographics

The mean age was 48.8 years (SD ± 17.5) for the whole studied population (548 patients). Of the patients, 57.7% (n = 316) were >45 years old, and most were female (n = 382, 69.7%). Positivity for either IgM or IgG serology (CHIKV confirmation) was 53.8% (n = 295) from the total 548 evaluated patients (Table 1). Of those, 6.8% (n = 20) were IgM positive, 71.9% (n = 212) were IgG positive and 21.3% (n = 63) were both IgM and IgG positive. According to the WHO criteria for acute clinical CHIKV infection, only 50.5% (n = 149) of the patients were confirmed for the disease by serological analysis. 

### 3.3. Clinical Characteristics

**Figure 4:**
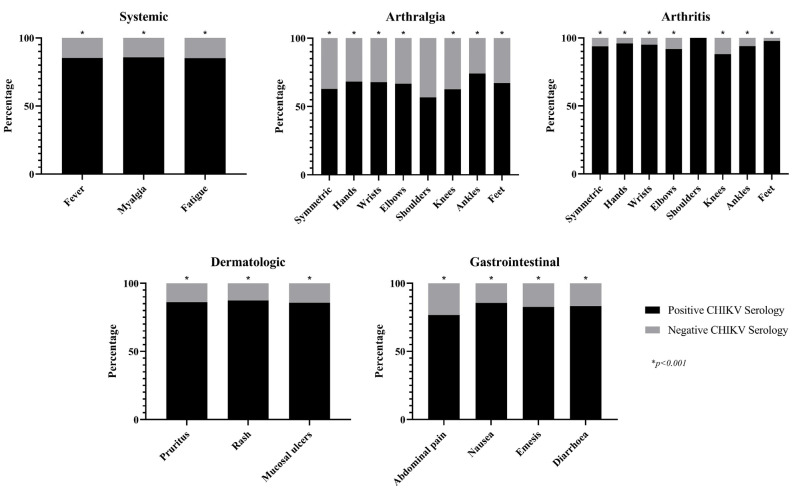
Clinical characteristics of the study population. CHIKV: Chikungunya virus.

In general, all clinical characteristics, including signs and symptoms found by the examiner or described by the patient, were more frequent in patients with serologically confirmed CHIKV infections (Figure 4). Notably, arthritis (regardless of the affected joint) was the most frequent symptom in this group of patients.

### 3.4. Univariate Analysis

After univariate analysis of signs and symptoms, only shoulder arthralgia was found with no statistical significance between patients with positive and negative CHIKV serology results (Table 2). Feet arthritis showed the highest odds ratio (OR: 45.4); however, it had the widest confidence interval (95% CI: 6.2–332.0). The best variables considering high OR and narrow CI were symmetric arthritis (OR: 18.1; 95% CI: 7.8–42.1), ankle arthritis (OR: 15.8; 95% CI: 4.8–51.4), abdominal rash (OR: 14.0; 95% CI: 6.3–31.0), and fatigue (OR: 10.5; 95% CI: 6.7–16.5).

### 3.5. Agreement and Expert Consensus Results

A series of questions were formulated for a group of specialists with statistically significant variables from the univariate analysis to evaluate agreement or disagreement in the diagnosis of CHIKV infection. There was disagreement on the following variables as clinical criteria: mucosal and gastrointestinal symptoms, shoulder and elbow arthralgia, and arthritis (Table 3).

### 3.6. Multiple Logistic Regression Analysis and ROC Curve

A multiple logistic regression model was used on the agreed variables to compare confirmed CHIKV-positive and CHIKV-negative patients. After the four steps in the model, CHIKV infection confirmed by positive serology result was independently associated with symmetric arthritis, rash, ankle joint pain, and fatigue. Each sign and symptom was assigned a point based on their coefficients (Table 4).

**Figure 5:**
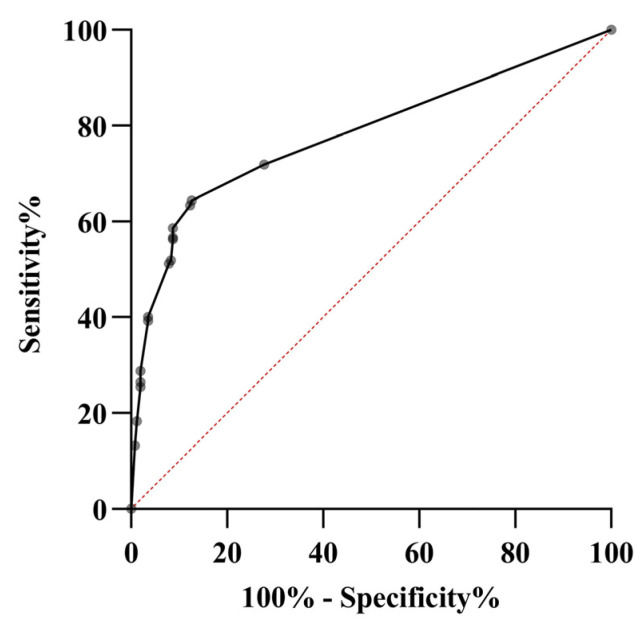
ROC curve of the screening score for CHIKV infection. ROC: Receiver operating characteristic; CHIKV: Chikungunya virus.

The ROC curve identified a cut-off value of 5.5, which maximized sensitivity and specificity (Figure 5). A score ≥ 5.5 was considered positive for identifying CHIKV-infected patients with a sensitivity of 64.4% (95% CI: 58.7–69.9%) and a specificity of 87.4% (95% CI: 82.7–91.2%). Based on this, we proposed a diagnostic screening clinical tool that consists of symmetric arthritis (4 points), fatigue (3 points), rash (2 points) and ankle joint pain (1 point).

**Figure 6:**
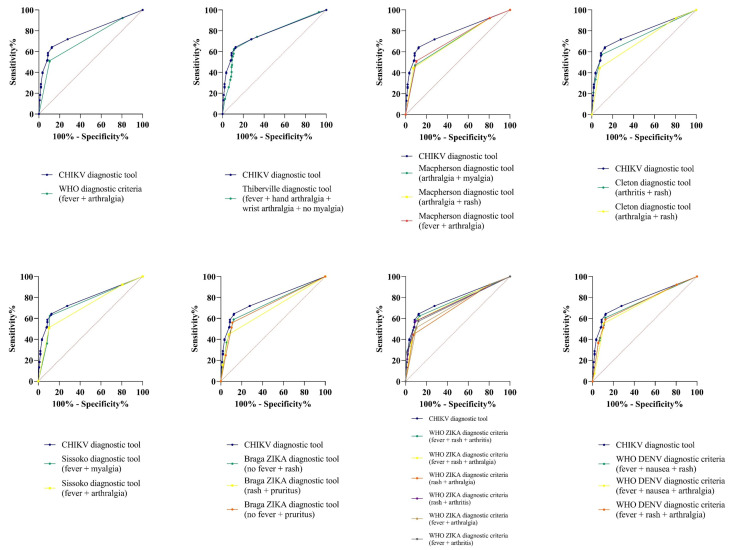
ROC curves of multiple diagnostic and screening tools in CHIKV, DENV and ZIKAV. ROC: Receiver operating characteristic; CHIKV: Chikungunya virus, DENV: Dengue virus; ZIKAV: Zika virus.

Previously reported clinical diagnostic and screening tools for CHIKV and other arboviral infections were applied to our cohort of patients to evaluate the performance in terms of sensibility, specificity, positive predictive value, negative predictive value, area under the curve, accuracy, and Youden index (Figure 6). The definitions of each screening tool are described in Supplementary Table S3. In a nutshell, the presence of the following symptoms for each diagnostic tool were considered as positive for CHIKV infection: CHIKV WHO case definition (fever plus arthralgia), Sissoko’s CHIKV screening tool (fever plus arthralgia or fever plus myalgia), Thiberville’s CHIKV screening tool (fever plus hand arthralgia plus wrist arthralgia plus absence of myalgia), Cleton’s syndromic approach for CHIKV (arthritis plus rash), Macpherson’s CHIKV screening tool(arthralgia plus myalgia, or arthralgia plus rash, or arthralgia plus fever), ZIKAV WHO case definition (rash plus fever plus arthralgia, or rash plus fever plus arthritis, or rash plus arthralgia, or rash plus arthritis, or fever plus arthralgia, or fever plus arthritis), Braga’s ZIKAV screening tool (absence of fever plus rash, or rash plus pruritus, or absence of fever plus pruritus) and DENV WHO case definition (fever plus nausea plus rash, or fever plus nausea plus arthralgia, or fever plus rash plus arthralgia). Our score results were compared to the other established clinical diagnostic tools to evaluate its performance (Table 5).

## 4. Discussion

In the present study, we analyzed the clinical signs and symptoms of 548 patients with suspected CHIKV infection and their association with confirmed CHIKV serology results to formulate a clinical screening tool for use in primary care settings. After univariate analysis, the variables associated with positive CHIKV serology results were discussed with an expert panel. Based on their experience in diagnosis and disease treatment, the most representative variables in CHIKV-infected patients were established.

It is well known that expert consensus defines the most appropriate selection of variables by considering different perspectives and positions of the experts consulted in the process [43]. Therefore, we included more meaningful and valuable variables for clinicians in the final multivariate analysis. The symptoms independently associated with CHIKV infection were observed to be symmetric arthritis, fatigue, rash, and ankle joint pain. A clinical screening tool was developed, which yielded high specificity (87.4%) and positive predictive value (PPV; 85.6%) with moderate sensitivity (64.4%) and negative predictive value (NPV; 67.8%).

When assessing people´s health, two types of tests are used: diagnostic tests that offer final information on the presence or absence of a condition and screening tests that are less demanding on the healthcare system, more accessible, and less invasive, time-consuming, and expensive [44]. The screening tests display ideal characteristics for countries where arboviral diseases caused by CHIKV, DENV, and ZIKAV are endemic. These tests are evaluated according to their sensitivity, specificity, PPV, and NPV. In brief, sensitivity and specificity refer to the accuracy of a screening test with a reference or gold standard, whereas PPV and NPV indicate the success of a screening test in classifying people as having or not having a condition [44]. Therefore, in screening situations for individuals in a clinical setting, it is more appropriate to use the PPVs and NPVs for evaluating the performance of a screening tool. Our screening tool has a high PPV but a moderate NPV. A high PPV is desirable in situations where the costs of diagnostics, treatment, and services are increased when the studied condition progresses slowly or is not life-threatening [44]. A moderate NPV might be acceptable if later assessments can be programmed and completed or if the condition is possible to sort out with no treatment [44]. Since chikungunya disease displays the above-mentioned characteristics, we believe that our screening tool will be useful in diagnosing CHIKV infection in a primary care setting where only clinical variables are at hand.

Other performance indicators of our screening tool, such as the Youden index (YI; 52) and accuracy (75.0%) when applied to our cohort, were higher than the previously developed diagnostic criteria or screening tools for CHIKV infection. The WHO case definition showed lower performance values in our cohort than our screening tool. Due to the ambiguity of the definition (Supplementary Table S3), only the mandatory symptoms (joint pain and fever) were used to calculate performance in our cohort [31]. Fever and joint pain are commonly included in other arboviral case definitions. For example, the WHO ZIKAV case definition (2016) states that the presence of fever or rash plus at least one more symptom, of which arthralgia is one, constitutes a suspected case [41]. In addition, the WHO DENV case definition includes fever plus two more symptoms, among which pain and aches are also present [42]. In fact, the recurring symptoms in the WHO case definitions of CHIKV, ZIKAV, and DENV are fever, aches, and pain (interpreted as arthralgia or myalgia). Furthermore, in the WHO CHIKV case definition, the use of terms such as “usually incapacitating” or “usually accompanied by…” could lead to misinterpretation by physicians, resulting in over- or underdiagnosis and, subsequently, poor performance when used in epidemics. Moreover, using fever as a mandatory or inclusion symptom dismisses asymptomatic patients, which increases the percentage of false negatives and selection bias.

Multiple attempts have been made to develop a better screening tool. Sissoko et al. (2010) found the combination of fever and polyarthralgia as the most relevant clinical pattern of CHIKV infection to identify presumptive cases during epidemics, yielding an accuracy of 87% with high sensitivity (84%) and specificity (89%) [11]. However, when applied to our cohort, the accuracy and sensitivity decreased to 69% and 51%, respectively. A possible explanation could be attributed to the median age of their cohort (24 years) since symptomatic expression of infection is lower in younger age groups [11].

In 2013, Thiberville et al. developed a clinical score with fever and arthralgia as mandatory symptoms. They added the presence of specific joint involvement (wrist or hand arthralgia) and the absence of myalgia to improve performance [26]. Their clinical score had the best performance in our cohort, with similar results as our own (Table 5). We believe that the resemblance lies in the use of specific disease symptoms. Our screening tool requires the inclusion of symmetrical arthritis or ankle joint pain to reach the cut-off point when added to other more generic symptoms such as fatigue and rash. These symptoms are almost unique to CHIKV infection and are rare in other arboviral infections caused by ZIKAV or DENV.

A study by Macpherson et al. (2014) found that a patient with joint pain and any combination of fever, myalgia, or rash was in 85% agreement with a positive CHIKV serological test result [12]. However, when applied to our cohort, the combination of arthralgia and fever yielded the best accuracy but sacrificed YI. Combining arthralgia with myalgia or rash increased specificity at the expense of sensitivity.

Other authors have elaborated on screening tools using simple clinical laboratory parameters. For example, the performance of the Thiberville screening tool increases if lymphopenia is present [26]. Godaert et al. (2017) used lymphopenia in the presence of fever and ankle arthralgia and the absence of neutrophil leukocytosis for CHIKV infection screening in elderly people [16]. Laboratory studies improve diagnosis; however, even simple laboratory tests are sometimes unavailable to primary care physicians. Therefore, developing a diagnostic tool based on clinical parameters was our primary goal.

With the appearance of ZIKAV epidemics, the clinical symptoms that help differentiate CHIKV from ZIKAV or DENV have been studied. Cleton et al. (2015) found that arthralgia, arthritis, and rash were associated with CHIKV infection, whereas DENV-positive patients had increased odds ratios for rash, fever, and hemorrhagic symptoms [40]. In our cohort, the syndromic combination of arthritis and rash yielded a high specificity but moderate to low sensitivity, yet showed a similar PPV and NPV to our screening tool.

Sahadeo et al. (2015) compared patients with confirmed DENV and CHIKV infections to obtain clinical and laboratory features that could help distinguish between the two diseases [27]. The combination with the best performance in differentiating between DENV and CHIKV infection was rash, joint pain, and leukocyte count <7 × 10^3^/µL. However, the PPV (58%) was less than optimal.

Another study by Lee et al. (2012) designed decision tree models for discriminating between DENV and CHIKV infections using clinical symptoms (presence of fever and its duration, bleeding, and illness) or laboratory tests (presence of thrombocytopenia) [22]. Interestingly, fever was associated with DENV infection and absent in CHIKV infection. A similar feature was found in our screening tool, where fever was not a mandatory symptom for suspected CHIKV diagnosis.

In a noteworthy study by Braga et al. (2017), a case definition was developed from a cohort of patients where CHIKV, DENV, and ZIKA were co-circulating [17]. A score ≥ 7.5 resulted in the diagnosis of ZIKAV from the following symptoms: pruritus, rash, conjunctival hyperemia, and the absence of fever and anorexia. This is consistent with the decision tree of Lee et al. (2012) and our screening tool, where fever as a symptom was not included. Half of the patients in our cohort with confirmed CHIKV would test positive for ZIKAV according to Braga’s ZIKAV case definition using a combination of no fever and rash (true positives: 58.9%), rash and pruritus (true positives: 44.7%), and no fever or pruritus (true positives: 55.9%). This can be explained by the fact that rash, a frequent symptom in our CHIKV-confirmed patients (87.4%), was awarded a high score (7 points from a cut-off of 7.5) in Braga’s case definition. Other studies on CHIKV epidemics have reported similar findings [45,46]. One could argue that if the CHIKV sample in Braga et al. (2017) study was larger, the appointed value for rash would be smaller and would have less preponderance in the score.

**Figure 7:**
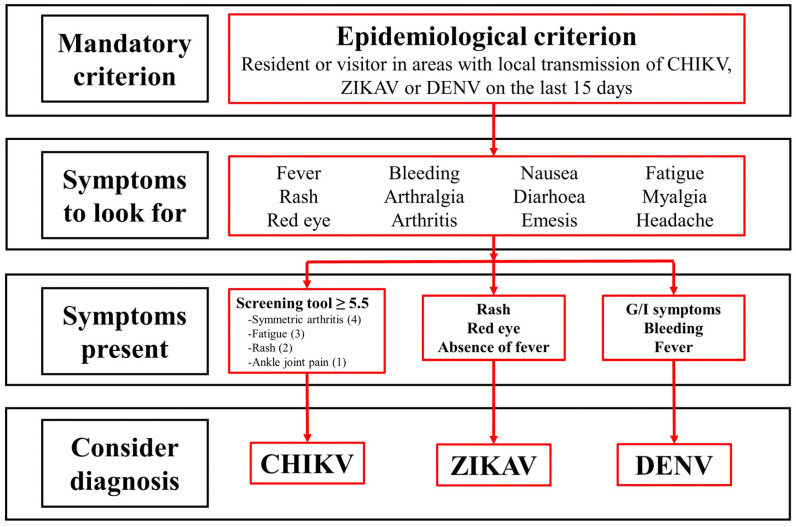
Proposed algorithm for clinical approach of CHIKV, ZIKAV and DENV. DENV: Dengue virus; CHIKV: Chikungunya virus; ZIKAV: Zika virus.

The same exercise yielded similar results when the WHO DENV case definitions were applied to our cohort. This exemplifies the need to assign importance to cardinal and specific symptoms of each infection. Although fatigue and rash are present in other definitions of DENV and ZIKAV, the presence of joint involvement (symmetrical arthritis or ankle joint pain) is mandatory in our screening tool to reach the cut-off point of 5.5. With this in mind, we proposed an algorithm for the clinical approach to CHIKV, ZIKAV, and DENV infections (Figure 7).

Our study has some limitations. First, given that our study resulted from the structure of a COPCORD approach, there is a selection bias regarding MSK symptoms. Second, since there was no physical exam when the symptoms started in each patient, these symptoms could not be validated by a physician; therefore, recall bias could be present. At the same time, since subjects were evaluated in a house-to-house system and not in an emergency room or primary care setting, the chance of finding acute and severe cases is reduced. This explains the low percentage of patients with CHIKV IgM-positive serology. Third, PCR was not performed to confirm CHIKV infection because of its high cost. Lastly, our screening tool has not been validated in other cohorts.

Nevertheless, our study has several strengths. The country’s population is well represented in the study, bearing in mind the number of samples and the six geographic regions in which the study was performed. In addition, the accuracy of physical examination, especially of the musculoskeletal system, is assured because all the patients were evaluated by a trained or in training professional of rheumatology. Finally, since the patients were evaluated in their homes and not in a medical setting, we could find even asymptomatic patients who otherwise would not visit a physician.

## 5. Conclusions

We developed a screening tool for CHIKV diagnosis using only clinical symptoms and proposed an algorithm to aid primary care physicians in the approach to common arboviral infections when laboratory tests are not available.

## Figures and Tables

**Table 1:** Demographics in patients with suspected CHIKV infection.

	Positive CHIKV Serology † (n = 295)	Negative CHIKV Serology ‡ (n = 253)	*p* Value
**Age in Years (mean ± SD)**	48.3 ± 17.4	49.6 ± 17.6	
**Gender**			
** ** **Female**	208 (70.5%)	174 (68.8%)	
** ** **Male**	87 (29.5%)	79 (31.2%)	
**Age-group in Years**			
** ** **18–29**	60 (20.3%)	37 (14.6%)	
** ** **30–39**	43 (14.6%)	47 (18.6%)	
** ** **40–49**	42 (14.2%)	45 (17.8%)	
** ** **50–59**	60 (20.3%)	44 (17.4%)	
** ** **60–69**	56 (19.0%)	44 (17.4%)	
** ** **70–79**	26 (8.8%)	25 (9.9%)	
** ** **≥80**	8 (2.7%)	11 (4.3%)	
**WHO acute clinical case** [31]			
** ** **Fulfill criteria**	149 (50.5%)	26 (10.3%)	<0.001
** ** **Do not fulfill criteria**	146 (49.5%)	227 (89.7%)	<0.001

CHIKV: Chikungunya virus; † IgM- or IgG-positive CHIKV serology; ‡ IgM- or IgG-negative CHIKV serology; WHO: World Health Organization Criteria for confirmed case of CHIKV; SD: Standard deviation.

**Table 2:** Univariate analysis of clinical features of patients with suspected CHIKV infection.

	Positive CHIKV Serology † (n = 295)	Negative CHIKV Serology ‡ (n = 253)	Total (n = 548)	OR (CI)	*p* Value
**Systemic**					
Fever	151 (85.3%)	26 (14.7%)	177 (32.3%)	9.1 (5.7–14.6)	<0.001
Myalgia	139 (85.8%)	23 (14.2%)	162 (29.6%)	8.9 (5.5–14.5)	<0.001
Whole body	40 (95.2%)	2 (4.8%)	42 (7.7%)	19.7 (4.7–82.3)	<0.001
Extremities	96 (82.1%)	21 (17.9%)	117 (21.4%)	5.3 (3.2–8.9)	<0.001
Back	22 (84.6%)	4 (15.4%)	26 (4.7%)	5.0 (1.7–14.7)	0.001
Fatigue	173 (85.2%)	30 (14.8%)	203 (37.0%)	10.5 (6.7–16.5)	<0.001
**Joint**					
Arthralgia	270 (57.0%)	204 (43.0%)	474 (86.5%)	2.6 (1.5–4.3)	<0.001
Symmetric	240 (62.8%)	142 (37.2%)	382 (69.7%)	3.4 (2.3–5.0)	<0.001
Hands	158 (68.4%)	73 (31.6%)	231 (42.2%)	2.8 (1.9–4.0)	<0.001
Wrists	93 (67.9%)	44 (32.1%)	137 (25.0%)	2.1 (1.4–3.3)	<0.001
Elbows	74 (66.7%)	37 (33.3%)	111 (20.3%)	1.9 (1.3–3.0)	0.002
Shoulders	81 (56.6%)	62 (43.4%)	143 (26.1%)	1.2 (0.8–1.7)	0.433
Knees	184 (62.6%)	110 (37.4%)	294 (53.6%)	2.1 (1.5–3.0)	<0.001
Ankles	137 (74.1%)	48 (25.9%)	185 (33.8%)	3.7 (2.5–5.4)	<0.001
Feet	104 (67.1%)	51 (32.9%)	155 (28.3%)	2.2 (1.5–3.2)	<0.001
Arthritis	99 (91.7%)	9 (8.3%)	108 (19.7%)	13.7 (6.7–27.8)	<0.001
Symmetric	90 (93.8%)	6 (6.3%)	96 (17.5%)	18.1 (7.8–42.1)	<0.001
Hands	47 (95.9%)	2 (4.1%)	49 (8.9%)	23.8 (5.7–99.0)	<0.001
Wrists	19 (95.0%)	1 (5.0%)	20 (3.6%)	17.3 (2.3–130.5)	<0.001
Elbows	11 (91.7%)	1 (8.3%)	12 (2.2%)	9.7 (1.2–76.1)	0.008
Shoulders	9 (100.0%)	0 (0.0%)	9 (1.6%)		
Knees	22 (88.0%)	3 (12.0%)	25 (4.6%)	6.7 (2.0–22.7)	<0.001
Ankles	47 (94.0%)	3 (6.0%)	50 (9.1%)	15.8 (4.8–51.4)	<0.001
Feet	45 (97.8%)	1 (2.2%)	46 (8.4%)	45.4 (6.2–332.0)	<0.001
**Dermatologic**					
Rash	132 (87.4%)	19 (12.6%)	151 (27.6%)	9.9 (6.0–16.8)	<0.001
Face	94 (88.7%)	12 (11.3%)	106 (19.3%)	9.4 (5.0–17.6)	<0.001
Thorax	84 (91.3%)	8 (8.7%)	92 (16.8%)	12.2 (5.8–25.6)	<0.001
Abdomen	84 (92.3%)	7 (7.7%)	91 (16.6%)	14.0 (6.3–31.0)	<0.001
Back	73 (91.3%)	7 (8.7%)	80 (14.6%)	11.5 (5.2–25.6)	<0.001
Extremities	91 (86.7%)	14 (13.3%)	105 (19.2%)	7.6 (4.2–13.7)	<0.001
Pruritus	87 (86.1%)	14 (13.9%)	101 (18.4%)	7.1 (3.9–13.0)	<0.001
Mucosa	12 (85.7%)	2 (14.3%)	14 (2.6%)	5.3 (1.2–24.0)	0.015
Oral	9 (90.0%)	1 (10.0%)	10 (1.8%)	7.9 (1.0–63.0)	0.021
Genital	11 (84.6%)	2 (15.4%)	13 (2.4%)	4.9 (1.1–22.1)	0.024
**Gastrointestinal**					
Diarrhea	75 (83.3%)	15 (16.7%)	90 (16.4%)	5.4 (3.0–9.6)	<0.001
Emesis	33 (82.5%)	7 (17.5%)	40 (7.3%)	4.4 (1.9–10.1)	<0.001
Nausea	34 (85.0%)	6 (15.0%)	40 (7.3%)	5.6 (2.2–13.0)	<0.001
Abdominal pain	23 (76.7%)	7 (23.3%)	30 (5.5%)	3.0 (1.2–7.0)	0.010

CHIKV: Chikungunya virus; † IgM- or IgG-positive CHIKV serology; ‡ IgM- or IgG-negative CHIKV serology; OR: Odds ratio; CI: 95% confidence interval.

**Table 3:** Agreement percentage to formulated questions on CHIKV clinical characteristics.

Do You Consider as Clinical Criteria?	Totally Agree	Agree	Not Agree or Disagree	Disagree	Totally Disagree	Type of Agreement (Total)
Symmetrical joint involvement	100	0	0	0	0	Agree (100)
Abrupt onset of symptoms	100	0	0	0	0	Agree (100)
Fever	38	50	12	0	0	Agree (78)
Rash	13	75	0	12	0	Agree (88)
Mucosal involvement	0	0	0	63	37	Disagree (100)
Myalgia	25	75	0	0	0	Agree (100)
Fatigue	63	25	12	0	0	Agree (88)
Gastrointestinal involvement	0	12	0	25	63	Disagree (88)
Shoulder arthralgia	0	25	12	38	25	Disagree (63)
Shoulder arthritis	0	0	0	38	62	Disagree (100)
Elbow arthralgia	0	0	0	88	12	Disagree (100)
Elbow arthritis	0	0	0	25	75	Disagree (100)
Wrist arthralgia	50	25	13	0	12	Agree (75)
Wrist arthritis	75	13	0	12	0	Agree (88)
Hand arthralgia	88	12	0	0	0	Agree (100)
Hand arthritis	88	12	0	0	0	Agree (100)
Knee arthralgia	13	63	0	12	12	Agree (76)
Knee arthritis	13	63	12	0	12	Agree (76)
Ankle arthralgia	100	0	0	0	0	Agree (100)
Ankle arthritis	100	0	0	0	0	Agree (100)
Foot arthralgia	50	38	0	12	0	Agree (88)
Foot arthritis	75	13	0	12	0	Agree (88)

CHIKV: Chikungunya virus.

**Table 4:** Multivariate analysis of predictors of CHIKV infection.

	Odds Ratio	95% Confidence Interval	*p* Value	Point Value
Symmetric arthritis	4.75	1.88–11.98	0.001	4
Fatigue	3.47	1.91–6.32	<0.001	3
Rash	2.70	1.37–5.31	0.004	2
Ankle joint pain	1.69	1.06–2.68	0.026	1

CHIKV: Chikungunya virus.

**Table 5:** Performance of different diagnostic tools in our confirmed CHIKV infected patients.

	Sensitivity % (CI)	Specificity % (CI)	PPV % (CI)	NPV % (CI)	AUC (CI)	Accuracy % (CI)	YI %
Proposed screening tool (Score ≥ 5.5)	64.4 (58.5–69.8)	87.3 (82.6–91.1)	85.5 (80.9–89.2)	67.7 (64.1–71.1)	0.72 (0.67–0.76)	75.0 (71.1–78.5)	52
CHIKV WHO case definition (2015) [31] Fever + arthralgia	51.2 (45.3–57.0)	85.3 (85.3–93.1)	85.3 (79.9–89.5)	61.2 (58.2–64.1)	0.71 (0.67–0.75)	68.9 (64.9–72.8)	36
Sissoko (2010) [11]							
Fever + arthralgia	51.2	85.3	85.3	61.2	0.71	68.9	36
	(45.3–57.0)	(85.3–93.1)	(79.9–89.5)	(58.2–64.1)	(0.67–0.75)	(64.9–72.8)	
Fever + myalgia	62.3	88.9	86.7	66.9	0.75	74.6	51
	(56.5–67.9)	(84.4–92.5)	(82.1–90.4)	(63.4–70.2)	(0.71–0.79)	(70.7–78.2)	
Thiberville (2013) [26]							
Fever + arthralgia hands +	62.3 (56.5–67.9)	88.9 (84.4–92.5)	86.7 (82.1–90.4)	66.9 (63.4–70.2)	0.76 (0.72–0.81)	74.6 (70.7–78.2)	51
arthralgia wrists +							
no myalgia							
Cleton syndromic approach (2015) [40]	57.2	90.5	87.5	64.5	0.74	72.6	48
Arthritis + rash	(51.4–63.0)	(86.2–93.8)	(82.6–91.2)	(61.2–67.6)	(0.70–0.78)	(68.6–76.3)	
Macpherson (2016) [12]							
Arthralgia + myalgia	47.1	90.9	85.8	59.6	0.71	67.3	38
	(41.3–52.9)	(86.6–94.1)	(80.1–90.1)	(56.8–62.3)	(0.66–0.74)	(63.2–71.2)	
Arthralgia + rash	44.7	92.4	87.4	58.9	0.70	66.7	38
	(38.9–50.6)	(88.5–95.4)	(81.5–91.6)	(56.2–61.5)	(0.66–0.74)	(62.6–70.7)	
Arthralgia + fever	51.2	85.3	85.3	61.2	0.71	68.9	36
	(45.3–57.0)	(85.3–93.1)	(79.9–89.5)	(58.2–64.1)	(0.67–0.75)	(64.9–72.8)	
ZIKAV WHO case definition (2016) [41]							
Rash + fever + arthralgia	58.8	87.7	84.8	64.7	0.74	72.2	46
	(53.1–64.6)	(83.1–91.5)	(79.9–88.7)	(61.3–67.9)	(0.70–0.78)	(68.3–75.9)	
Rash + fever + arthritis	62.3	86.5	84.4	66.3	0.76	73.5	49
	(56.5–67.9)	(81.7–90.5)	(79.6–88.2)	(62.8–69.7)	(0.72–0.80)	(69.6–77.1)	
Rash + arthralgia	44.7	92.4	87.4	58.9	0.70	66.7	38
	(38.9–50.6)	(88.5–95.4)	(81.5–91.6)	(56.2–61.5)	(0.66–0.74)	(62.6–70.7)	
Rash + arthritis	57.2	90.5	87.5	64.5	0.74	72.6	48
	(51.4–63.0)	(86.2–93.8)	(82.6–91.2)	(61.2–67.6)	(0.70–0.78)	(68.6–76.3)	
Fever + arthralgia	51.2	85.3	85.3	61.2	0.71	68.9	36
	(45.3–57.0)	(85.3–93.1)	(79.9–89.5)	(58.2–64.1)	(0.67–0.75)	(64.9–72.8)	
Fever + arthritis	57.2	87.7	84.5	63.7	0.73	71.3	45
	(51.4–63.0)	(83.1–91.5)	(79.4–88.4)	(60.5–66.9)	(0.69–0.77)	(67.3–75.1)	
Braga ZIKAV (2017) [17]							
No fever + rash	58.9	87.7	84.8	64.7	0.74	72.2	47
	(53.1–64.6)	(83.1–91.5)	(79.9–88.7)	(61.3–67.9)	(0.69–0.78)	(68.3–75.9)	
Rash + pruritus	44.7	92.4	87.4	58.9	0.68	66.7	37
	(38.9–50.6)	(88.5–95.4)	(81.5–91.6)	(56.2–61.5)	(0.64–0.73)	(62.6–70.7)	
No fever + pruritus	55.9	88.9	85.4	63.3	0.72	71.1	45
	(50.0–61.6)	(84.4–92.5)	(80.3–89.4)	(60.1–66.4)	(0.68–0.76)	(67.1–74.9)	
DENV WHO case definition (2009) [42]							
Fever + nausea + rash	60.7	87.7	85.2	65.6	0.74	73.1	48
	(54.8–66.2)	(83.1–91.5)	(80.3–89.0)	(62.2–68.9)	(0.70–0.78)	(69.2–76.8)	
Fever + nausea + arthralgia	55.2	89.3	85.7	63.1	0.72	70.9	45
	(49.3–61.0)	(84.8–92.8)	(80.6–89.7)	(59.9–66.1)	(0.68–0.77)	(66.9–74.7)	
Fever + rash + arthralgia	58.8	87.7	84.8	64.7	0.74	72.2	46
	(53.1–64.6)	(83.1–91.5)	(79.9–88.7)	(61.3–67.9)	(0.70–0.78)	(68.3–75.9)	

CHIKV: Chikungunya virus; CI: 95% confidence interval; PPV: Positive predictive value; NPV: Negative predictive value; AUC: Area under the curve; YI: Youden’s index (Sensitivity + sensibility − 100); WHO: World Health Organization; ZIKAV: Zika virus; DENV: Dengue virus.

## Data Availability

The data that support the findings of this study are available from the corresponding author, J.L., upon reasonable request.

## Supplementary Materials

The following supporting information can be downloaded at: https://www.mdpi.com/article/10.3390/tropicalmed8040213/s1, Table S1: Percentage of most common symptoms in arboviral diseases; Table S2: Statistical differences between symptoms and arboviral diseases; Table S3: Definitions of diagnostic criteria and screening tools.

## References

[1] Powers A.M., Brault A.C., Tesh R.B., Weaver S.C. (2000). Re-emergence of chikungunya and o’nyong-nyong viruses: Evidence for distinct geographical lineages and distant evolutionary relationships. J. Gen. Virol..

[2] Tsetsarkin K.A., Vanlandingham D.L., McGee C.E., Higgs S. (2007). A single mutation in Chikungunya virus affects vector specificity and epidemic potential. PLoS Pathog..

[3] The Pan American Health Organization (2015). Number of Reported Cases of Chikungunya Fever in the Americas, by Country or Territory Cumulative Cases (Updated 15 May 2015) Data Source: Cases Reported by IHR NFPs to PAHO/WHO and/or through Member States Websites or Official News Publication.

[4] Padilla J.C., Lizarazo F.E., Murillo O.L., Mendigaña F.A., Pachón E., Vera M.J. (2017). Epidemiología de las principales enfermedades transmitidas por vectores en Colombia, 1990–2016. Biomédica.

[5] Rodas J.D., Kautz T., Camacho E., Paternina L., Guzmán H., Díaz F.J., Blanco P., Tesh R., Weaver S.C. (2016). Genetic Characterization of Northwestern Colombian Chikungunya Virus Strains from the 2014–2015 Epidemic. Am. J. Trop. Med. Hyg..

[6] Cassadou S., Boucau S., Petit-Sinturel M., Huc P., Leparc-Goffart I., Ledrans M. (2014). Emergence of chikungunya fever on the French side of Saint Martin island, October to December 2013. Eurosurveillance.

[7] Dengue Around the World|Dengue|CDC. https://www.cdc.gov/dengue/areaswithrisk/around-the-world.html.

[8] Geographic Distribution|Chikungunya Virus|CDC. https://www.cdc.gov/chikungunya/geo/index.html.

[9] Zika Travel Information|Travelers’ Health|CDC. https://wwwnc.cdc.gov/travel/page/zika-information.

[10] Kularatne S.A.M., Gihan M.C., Weerasinghe S.C., Gunasena S. (2009). Concurrent outbreaks of Chikungunya and Dengue fever in Kandy, Sri Lanka, 2006–2007: A comparative analysis of clinical and laboratory features. Postgrad. Med. J..

[11] Sissoko D., Ezzedine K., Moendandzé A., Giry C., Renault P., Malvy D. (2010). Field evaluation of clinical features during chikungunya outbreak in Mayotte, 2005–2006. Trop. Med. Int. Heal..

[12] Macpherson C., Noël T., Fields P., Jungkind D., Yearwood K., Simmons M., Widjaja S., Mitchell G., Noel D., Bidaisee S. (2016). Clinical and serological insights from the asian lineage Chikungunya outbreak in Grenada, 2014: An observational study. Am. J. Trop. Med. Hyg..

[13] Bloch D., Roth N.M., Caraballo E.V., Muñoz-Jordan J., Hunsperger E., Rivera A., Pérez-Padilla J., Rivera Garcia B., Sharp T.M. (2016). Use of Household Cluster Investigations to Identify Factors Associated with Chikungunya Virus Infection and Frequency of Case Reporting in Puerto Rico. PLoS Negl. Trop. Dis..

[14] van Genderen F.T., Krishnadath I., Sno R., Grunberg M.G., Zijlmans W., Adhin M.R. (2016). First Chikungunya Outbreak in Suriname; Clinical and Epidemiological Features. PLoS Negl. Trop. Dis..

[15] Anaya J.-M., Rodríguez Y., Monsalve D.M., Vega D., Ojeda E., González-Bravo D., Rodríguez-Jiménez M., Pinto-Díaz C.A., Chaparro P., Gunturiz M.L. (2017). A comprehensive analysis and immunobiology of autoimmune neurological syndromes during the Zika virus outbreak in Cúcuta, Colombia. J. Autoimmun..

[16] Godaert L., Bartholet S., Najioullah F., Hentzien M., Fanon J.L., Césaire R., Dramé M. (2017). Screening for Chikungunya virus infection in aged people: Development and internal validation of a new score. PLoS ONE.

[17] Braga J.U., Bressan C., Dalvi A.P.R., Calvet G.A., Daumas R.P., Rodrigues N., Wakimoto M., Nogueira R.M.R., Nielsen-Saines K., Brito C. (2017). Accuracy of Zika virus disease case definition during simultaneous Dengue and Chikungunya epidemics. PLoS ONE.

[18] Silva M.M., Tauro L.B., Kikuti M., Anjos R.O., Santos V.C., Gonçalves T.S.F., Paploski I.A.D., Moreira P.S.S., Nascimento L.C.J., Campos G.S. (2019). Concomitant transmission of dengue, chikungunya and Zika viruses in Brazil: Clinical and epidemiological findings from surveillance for acute febrile illness. Clin. Infect. Dis..

[19] Carabali M., Lim J.K., Palencia D.C., Lozano-Parra A., Gelvez R.M., Lee K.S., Florez J.P., Herrera V.M., Kaufman J.S., Rojas E.M. (2018). Burden of dengue among febrile patients at the time of chikungunya introduction in Piedecuesta, Colombia. Trop. Med. Int. Heal..

[20] Sánchez-Carbonel J., Tantaléan-Yépez D., Aguilar-Luis M.A., Silva-Caso W., Weilg P., Vásquez-Achaya F., Costa L., Martins-Luna J., Sandoval I., del Valle-Mendoza J. (2018). Identification of infection by Chikungunya, Zika, and Dengue in an area of the Peruvian coast. Molecular diagnosis and clinical characteristics. BMC Res. Notes.

[21] Azeredo E.L., Hoscher Romanholi I., Badolato-Corrêa J., Cunha R., Barbosa L.S., de-Oliveira-Pinto L.M., Dal Fabbro M., dos Santos F.B., Sánchez-Arcila J.C., Nunes P.C.G. (2018). Clinical and Laboratory Profile of Zika and Dengue Infected Patients: Lessons Learned From the Co-circulation of Dengue, Zika and Chikungunya in Brazil. PLoS Curr..

[22] Lee V.J., Chow A., Zheng X., Carrasco L.R., Cook A.R., Lye D.C., Ng L.C., Leo Y.S. (2012). Simple Clinical and Laboratory Predictors of Chikungunya versus Dengue Infections in Adults. PLoS Negl. Trop. Dis..

[23] Vega F.L.R., Bezerra J.M.T., Said R.F.d.C., da Gama Neto A.N., Cotrim E.C., Mendez D., Amâncio F.F., Carneiro M. (2019). Emergence of chikungunya and Zika in a municipality endemic to dengue, Santa Luzia, MG, Brazil, 2015–2017. Rev. Soc. Bras. Med. Trop..

[24] Taraphdar D., Sarkar A., Mukhopadhyay B.B., Chatterjee S. (2012). A Comparative Study of Clinical Features between Monotypic and Dual Infection Cases with Chikungunya Virus and Dengue Virus in West Bengal, India. Am. J. Trop. Med. Hyg..

[25] Mohd Zim M.A., Sam I.-C., Omar S.F.S., Chan Y.F., AbuBakar S., Kamarulzaman A. (2013). Chikungunya infection in Malaysia: Comparison with dengue infection in adults and predictors of persistent arthralgia. J. Clin. Virol..

[26] Thiberville S.D., Boisson V., Gaudart J., Simon F., Flahault A., de Lamballerie X. (2013). Chikungunya Fever: A Clinical and Virological Investigation of Outpatients on Reunion Island, South-West Indian Ocean. PLoS Negl. Trop. Dis..

[27] Sahadeo N., Mohammed H., Allicock O.M., Auguste A.J., Widen S.G., Badal K., Pulchan K., Foster J.E., Weaver S.C., Carrington C.V.F. (2015). Molecular Characterisation of Chikungunya Virus Infections in Trinidad and Comparison of Clinical and Laboratory Features with Dengue and Other Acute Febrile Cases. PLoS Negl. Trop. Dis..

[28] Waggoner J.J., Gresh L., Vargas M.J., Ballesteros G., Tellez Y., Soda K.J., Sahoo M.K., Nuñez A., Balmaseda A., Harris E. (2016). Viremia and Clinical Presentation in Nicaraguan Patients Infected With Zika Virus, Chikungunya Virus, and Dengue Virus. Clin. Infect. Dis..

[29] Romero C., Zogbi H., Carvalho M.S., de Souza R.V., Calvet G.A., Brasil P., de Filippis A.M.B., Bressan C.d.S., de Mendonça M.C.L., Alves S.S. (2016). Zika Virus Outbreak in Rio de Janeiro, Brazil: Clinical Characterization, Epidemiological and Virological Aspects. PLoS Negl. Trop. Dis..

[30] Danis-Lozano R., Díaz-González E.E., Trujillo-Murillo K.d.C., Caballero-Sosa S., Sepúlveda-Delgado J., Malo-García I.R., Canseco-Ávila L.M., Salgado-Corsantes L.M., Domínguez-Arrevillaga S., Torres-Zapata R. (2017). Clinical characterization of acute and convalescent illness of confirmed chikungunya cases from Chiapas, S. Mexico: A cross sectional study. PLoS ONE.

[31] World Health Organization (WHO) (2015). Chikungunya: Case definitions for acute, atypical and chronic cases. Conclusions of an expert consultation, Managua, Nicaragua, 20–21 May. Relev. Epidemiol. Hebd..

[32] Petti C.A., Polage C.R., Quinn T.C., Ronald A.R., Sande M.A. (2006). Laboratory Medicine in Africa: A Barrier to Effective Health Care. Clin. Infect. Dis..

[33] Salas Botero D. Informe Final del Evento Chikungunya, Colombia 2014. https://www.ins.gov.co/buscador-eventos/Paginas/Info-Evento.aspx.

[34] Salas Botero D. Informe Final del Evento Chikungunya, Colombia 2015. https://www.ins.gov.co/buscador-eventos/Paginas/Info-Evento.aspx.

[35] Pinilla Farias A. Informe del Evento Chikungunya Periodo Epidemiológico XIII, Colombia 2016. https://www.ins.gov.co/buscador-eventos/Paginas/Info-Evento.aspx.

[36] Rodriguez Reyes A.J. Informe del Evento Chikungunya, Colombia 2017. https://www.ins.gov.co/buscador-eventos/Paginas/Info-Evento.aspx.

[37] Instituto Nacional de Salud (2018). Boletín Epidemiológico Semanal Semana Epidemiológica 52 23 al 29 de Diciembre de 2018.

[38] Instituto Nacional de Salud (2019). Semana Epidemiológica 44.

[39] Rueda J.C., Santos A.M., Angarita J.I., Giraldo R.B., Saldarriaga E.L., Ballesteros Muñoz J.G., Forero E., Valencia H., Somoza F., Martin-Arsanios D. (2019). Demographic and clinical characteristics of chikungunya patients from six Colombian cities, 2014–2015. Emerg. Microbes Infect..

[40] Fernández-Ávila D.G., Rojas M.X., Rosselli D. (2019). Delphi method in rheumatology research: Are we doing well?. Rev. Colomb. Reumatol..

[41] Trevethan R. (2017). Sensitivity, Specificity, and Predictive Values: Foundations, Pliabilities, and Pitfalls in Research and Practice. Front. Public Heal..

[42] OMS (2016). Zika virus disease: Interim case definitions. Who/Zikv/Sur/16.1 2016.

[43] World Health Organization (2009). Dengue: Guidelines for Diagnosis Treatment Prevention and Control (New Edition 2009).

[44] Cleton N.B., Reusken C.B.E.M., Wagenaar J.F.P., van der Vaart E.E., Reimerink J., van der Eijk A.A., Koopmans M.P.G. (2015). Syndromic Approach to Arboviral Diagnostics for Global Travelers as a Basis for Infectious Disease Surveillance. PLoS Negl. Trop. Dis..

[45] Borgherini G., Poubeau P., Staikowsky F., Lory M., Moullec N.L., Becquart J.P., Wengling C., Michault A., Paganin F. (2007). Outbreak of Chikungunya on Reunion Island: Early Clinical and Laboratory Features in 157 Adult Patients. Clin. Infect. Dis..

[46] Kumar R., Sharma M., Jain S., Yadav S., Singhal A. (2017). Cutaneous manifestations of chikungunya fever: Observations from an outbreak at a Tertiary Care Hospital in Southeast Rajasthan, India. Indian Dermatol. Online J..

