# Supplementary material for: Clinical Diagnosis of Chikungunya Infection: An Essential Aid in a Primary Care Setting Where Serological Confirmation Is Not Available
Source: Trop Med Infect Dis. 2023 Apr 3;8(4):213. doi: 10.3390/tropicalmed8040213 (PMC10146408; doi:10.3390/tropicalmed8040213)
Supplement: Supplementary file 1 [file tropicalmed-08-00213-s001.zip › tropicalmed-2283827-supplementary.pdf]

Tropical Medicine and Infectious Disease

MDPI

Supplementary Materials of Clinical Diagnosis of Chikungunya Infection: An Essential Aid in a Primary Care Setting Where Serological Confirmation is not Available

Table S1. Percentage of most common symptoms in arboviral diseases.

	DENV	CHIKV	ZIKAV	Reference
Fever	93.89	92.66	69.51	[10, 11, 20–29, 12, 30, 13-19]
Headache	73.33	64.14	72.19	[10, 11, 20-23, 25, 27-29, 12-19]
Rash	37.00	47.03	74.03	[10, 11, 20-23, 25-30, 12-19]
G/I symptoms	49.09	37.46	28.88	[10, 11, 20-23, 25-30, 12-19]
Myalgia	73.43	61.48	65.54	[10, 11, 21-29, 12-19]
Fatigue	12.50	43.78	40.55	[15, 16, 18, 22, 23, 27, 28]
Arthralgia	63.79	87.14	61.55	[10, 11, 20, 22, 23, 25-30, 12-19]
Arthritis/Oedema	11.50	48.36	27.80	[10, 13, 29, 18-23, 25, 27]
Bleeding	8.93	4.81	4.03	[10, 12, 14-17, 23, 28]
Red eye	34.38	29.42	62.04	[10, 13, 17-19, 21, 23, 29, 30]

DENV: Dengue virus; CHIKV: Chikungunya virus; ZIKAV: Zika virus.

Table S2. Statistical differences between symptoms and arboviral diseases.

	DENV-CHIKV	DENV-ZIKAV	CHIKV-ZIKAV
Fever		< 0.000	< 0.000
Headache			
Rash		< 0.000	< 0.000
G/I symptoms		0.0057	
Myalgia			
Fatigue	< 0.000	< 0.000	
Arthralgia	< 0.000		< 0.000
Arthritis/Oedema	< 0.000	0.003	0.005
Bleeding			
Red eye		< 0.000	< 0.000

DENV: Dengue virus; CHIKV: Chikungunya virus; ZIKAV: Zika virus.

Table S3. Definitions of diagnostic criteria and screening tools.

	Definition
CHIKV WHO case definition (2015) [31]	Acute clinical case: clinical criterion (fever ≥ 38.5 °C and joint pain usually incapacitating usually accompanied by exanthema, myalgia, back pain, and headache) and epidemiological criterion: resident or visitor in areas with local transmission of CHIKV on the last 15 days
Sissoko CHIKV screening tool (2010) [11]	Incapacitating polyarthralgia and fever of abrupt onset
Thiberville CHIKV screening tool (2013) [26]	Clinical score: fever and arthralgia for less than 48 hours plus: arthralgia of at least 1 MCP, arthralgia of at least 1 wrist and myalgia (probable CHIKV if 3 are present; possible CHIKV if only 2 are present)
Cleton CHIKV screening tool (2015) [40]	Arthralgia combined with rash (both symptoms present are associated with positive test outcome for CHIKV)
Macpherson CHIKV screening tool (2016) [12]	Joint pain and any combination of fever, body pain, or rash (the combination of symptoms that most closely agree with positive CHIKV serology)
ZIKAV WHO case definition (2016) [41]	Suspected case: a person presenting with rash and/or fever and at least one of the following signs or symptoms: arthralgia or arthritis or conjunctivitis (non-purulent/hyperaemic)
Braga ZIKAV screening tool (2017) [17]	Clinical based score to discriminate ZIKAV cases from other febrile or exanthematic illnesses (a score ≥ 7.5 from maculopapular rash = 7, temperature ≤ 37.5 °C = 6, itching = 4.5, anorexia = 2.5, no petechiae = 2, conjunctival hyperaemia = 1)

DENV WHO case definition (2009) [42]	Probable dengue: live in/travel to dengue endemic area. Fever and 2 of the following criteria: nausea or vomiting, rash, aches and pains, tourniquet test positive, leukopenia, any warning sign

CHIKV: Chikungunya virus; WHO: World Health Organization; ZIKAV: Zika virus; DENV: Dengue virus.

References

10. Kularatne, S.A.M.; Gihan, M.C.; Weerasinghe, S.C.; Gunasena, S. Concurrent outbreaks of Chikungunya and Dengue fever in Kandy, Sri Lanka, 2006-07: A comparative analysis of clinical and laboratory features. *Postgrad. Med. J.* **2009**, *85*, 342–346.
11. Sissoko, D.; Ezzedine, K.; Moendandzé, A.; Giry, C.; Renault, P.; Malvy, D. Field evaluation of clinical features during chikungunya outbreak in Mayotte, 2005-2006. *Trop. Med. Int. Heal.* **2010**, *15*, 600–607.
12. Macpherson, C.; Noël, T.; Fields, P.; Jungkind, D.; Yearwood, K.; Simmons, M.; Widjaja, S.; Mitchell, G.; Noel, D.; Bidaisee, S.; et al. Clinical and serological insights from the asian lineage Chikungunya outbreak in Grenada, 2014: An observational study. *Am. J. Trop. Med. Hyg.* **2016**, *95*, 890–893.
13. Bloch, D.; Roth, N.M.; Caraballo, E. V.; Muñoz-Jordan, J.; Hunsperger, E.; Rivera, A.; Pérez-Padilla, J.; Rivera Garcia, B.; Sharp, T.M. Use of Household Cluster Investigations to Identify Factors Associated with Chikungunya Virus Infection and Frequency of Case Reporting in Puerto Rico. *PLoS Negl. Trop. Dis.* **2016**, *10*, e0005075.
14. van Genderen, F.T.; Krishnadath, I.; Sno, R.; Grunberg, M.G.; Zijlmans, W.; Adhin, M.R. First Chikungunya Outbreak in Suriname; Clinical and Epidemiological Features. *PLoS Negl. Trop. Dis.* **2016**, *10*, 1–18.
15. Anaya, J.-M.; Rodríguez, Y.; Monsalve, D.M.; Vega, D.; Ojeda, E.; González-Bravo, D.; Rodríguez-Jiménez, M.; Pinto-Díaz, C.A.; Chaparro, P.; Gunturiz, M.L.; et al. A comprehensive analysis and immunobiology of autoimmune neurological syndromes during the Zika virus outbreak in Cúcuta, Colombia. *J. Autoimmun.* **2017**, *77*, 123–138.
16. Godaert, L.; Bartholet, S.; Najioullah, F.; Hentzien, M.; Fanon, J.L.; Césaire, R.; Dramé, M. Screening for Chikungunya virus infection in aged people: Development and internal validation of a new score. *PLoS One* **2017**, *12*, 1–9.
17. Braga, J.U.; Bressan, C.; Dalvi, A.P.R.; Calvet, G.A.; Daumas, R.P.; Rodrigues, N.; Wakimoto, M.; Nogueira, R.M.R.; Nielsen-Saines, K.; Brito, C.; et al. Accuracy of Zika virus disease case definition during simultaneous Dengue and Chikungunya epidemics. *PLoS One* **2017**, *12*, e0179725.
18. O Silva, M.M.; Tauro, L.B.; Kikuti, M.; Anjos, R.O.; Santos, V.C.; Gonçalves, T.S.F.; Paploski, I.A.D.; Moreira, P.S.S.; Nascimento, L.C.J.; Campos, G.S.; et al. Concomitant transmission of dengue, chikungunya and Zika viruses in Brazil: Clinical and epidemiological findings from surveillance for acute febrile illness. *Clin. Infect. Dis.* **2018**, *8*.
19. Carabali, M.; Lim, J.K.; Palencia, D.C.; Lozano-Parra, A.; Gelvez, R.M.; Lee, K.S.; Florez, J.P.; Herrera, V.M.; Kaufman, J.S.; Rojas, E.M.; et al. Burden of dengue among febrile patients at the time of chikungunya introduction in Piedecuesta, Colombia. *Trop. Med. Int. Heal.* **2018**, *23*, 1231–1241.
20. Sánchez-Carbonel, J.; Tantaléan-Yépez, D.; Aguilar-Luis, M.A.; Silva-Caso, W.; Weilg, P.; Vásquez-Achaya, F.; Costa, L.; Martins-Luna, J.; Sandoval, I.; del Valle-Mendoza, J. Identification of infection by Chikungunya, Zika, and Dengue in an area of the Peruvian coast. Molecular diagnosis and clinical characteristics. *BMC Res. Notes* **2018**, *11*, 175.
21. Azeredo, E.L.; Hoscher Romanholi, I.; Badolato-Corrêa, J.; Cunha, R.; Barbosa, L.S.; de-Oliveira-Pinto, L.M.; Dal Fabbro, M.; dos Santos, F.B.; Sánchez-Arcila, J.C.; Nunes, P.C.G.; et al. Clinical and Laboratory Profile of Zika and Dengue Infected Patients: Lessons Learned From the Co-circulation of Dengue, Zika and Chikungunya in Brazil. *PLoS Curr.* **2018**.
22. Lee, V.J.; Chow, A.; Zheng, X.; Carrasco, L.R.; Cook, A.R.; Lye, D.C.; Ng, L.C.; Leo, Y.S. Simple Clinical and Laboratory Predictors of Chikungunya versus Dengue Infections in Adults. *PLoS Negl. Trop. Dis.* **2012**, *6*.
23. Vega, F.L.R.; Bezerra, J.M.T.; Said, R.F. de C.; Gama Neto, A.N. da; Cotrim, E.C.; Mendez, D.; Amâncio, F.F.; Carneiro,

M. Emergence of chikungunya and Zika in a municipality endemic to dengue, Santa Luzia, MG, Brazil, 2015-2017. *Rev. Soc. Bras. Med. Trop.* **2019**, *52*, 1–9.
24. Taraphdar, D.; Sarkar, A.; Mukhopadhyay, B.B.; Chatterjee, S. A Comparative Study of Clinical Features between Monotypic and Dual Infection Cases with Chikungunya Virus and Dengue Virus in West Bengal, India. *Am. J. Trop. Med. Hyg.* **2012**, *86*, 720–723.
25. Mohd Zim, M.A.; Sam, I.-C.; Omar, S.F.S.; Chan, Y.F.; AbuBakar, S.; Kamarulzaman, A. Chikungunya infection in Malaysia: Comparison with dengue infection in adults and predictors of persistent arthralgia. *J. Clin. Virol.* **2013**, *56*, 141–145.
26. Thiberville, S.D.; Boisson, V.; Gaudart, J.; Simon, F.; Flahault, A.; de Lamballerie, X. Chikungunya Fever: A Clinical and Virological Investigation of Outpatients on Reunion Island, South-West Indian Ocean. *PLoS Negl. Trop. Dis.* **2013**, *7*.
27. Sahadeo, N.; Mohammed, H.; Allicock, O.M.; Auguste, A.J.; Widen, S.G.; Badal, K.; Pulchan, K.; Foster, J.E.; Weaver, S.C.; Carrington, C.V.F. Molecular Characterisation of Chikungunya Virus Infections in Trinidad and Comparison of Clinical and Laboratory Features with Dengue and Other Acute Febrile Cases. *PLoS Negl. Trop. Dis.* **2015**, *9*, 1–18.
28. Waggoner, J.J.; Gresh, L.; Vargas, M.J.; Ballesteros, G.; Tellez, Y.; Soda, K.J.; Sahoo, M.K.; Nuñez, A.; Balmaseda, A.; Harris, E.; et al. Viremia and Clinical Presentation in Nicaraguan Patients Infected With Zika Virus, Chikungunya Virus, and Dengue Virus. *Clin. Infect. Dis.* **2016**, *63*, 1584–1590.
29. Romero, C.; Zogbi, H.; Carvalho, M.S.; de Souza, R.V.; Calvet, G.A.; Brasil, P.; de Filippis, A.M.B.; Bressan, C. da S.; Mendonça, M.C.L. de; Alves, S.S.; et al. Zika Virus Outbreak in Rio de Janeiro, Brazil: Clinical Characterization, Epidemiological and Virological Aspects. *PLoS Negl. Trop. Dis.* **2016**, *10*, e0004636.
30. Danis-Lozano, R.; Díaz-González, E.E.; Trujillo-Murillo, K. del C.; Caballero-Sosa, S.; Sepúlveda-Delgado, J.; Malo-García, I.R.; Canseco-Ávila, L.M.; Salgado-Corsantes, L.M.; Domínguez-Arrevillaga, S.; Torres-Zapata, R.; et al. Clinical characterization of acute and convalescent illness of confirmed chikungunya cases from Chiapas, S. Mexico: A cross sectional study. *PLoS One* **2017**, *12*, 1–15.
31. World Health Organization (WHO) Chikungunya: case definitions for acute, atypical and chronic cases. Conclusions of an expert consultation, Managua, Nicaragua, 20-21 May 2015. *Relev. Epidemiol. Hebd.* **2015**, *90*, 410–4
40. Cleton, N.B.; Reusken, C.B.E.M.; Wagenaar, J.F.P.; van der Vaart, E.E.; Reimerink, J.; van der Eijk, A.A.; Koopmans, M.P.G. Syndromic Approach to Arboviral Diagnostics for Global Travelers as a Basis for Infectious Disease Surveillance. *PLoS Negl. Trop. Dis.* **2015**, *9*, 1–15.
41. OMS Zika virus disease: Interim case definitions. *Who/Zikv/Sur/16.1* **2016**, 2016.
42. Organization, W.H. *Dengue : Guidelines for Diagnosis Treatment Prevention and Control (New Edition 2009).*; World Health Organization, 2009; ISBN 978 92 4 154787 1.

Disclaimer/Publisher's Note: The statements, opinions and data contained in all publications are solely those of the individual author(s) and contributor(s) and not of MDPI and/or the editor(s). MDPI and/or the editor(s) disclaim responsibility for any injury to people or property resulting from any ideas, methods, instructions or products referred to in the content.
